# Temporal upregulation of TREM2 parallel to M2 macrophage marker expression in tuberculosis: implications for macrophage polarization regulation

**DOI:** 10.3389/fcimb.2026.1835965

**Published:** 2026-05-29

**Authors:** Xiaoqian Shang, Naifeisha Maimaiti, Fengming Tian, Chunbo Liao, Hu Sun, Xuan Zhou, Weina Kong, Qiannan Sun, Liang Wang, Xiumin Ma

**Affiliations:** 1 Clinical Laboratory Center, The third Affiliated Teaching Hospital of Xinjiang Medical University (Affiliated Cancer Hospital), Key Laboratory of High Incidence Diseases in Research in Xinjiang (Xinjiang Medical University), Ministry of Education, Urumqi, Xinjiang, China; 2 Department of Pathology, People's Hospital of Xinjiang Uygur Autonomous Region, Urumuqi, Xinjiang, China; 3 Clinical Laboratory Center, The Fifth Affiliated Hospital of Xinjiang Medical University, Urumqi, Xinjiang, China; 4 The Frist Affiliated Hospital of Xinjiang Medical University, Urumqi, Xinjiang, China; 5 The Fifth Affiliated Hospital of Xinjiang Medical University, Urumqi, Xinjiang, China

**Keywords:** macrophage, *Mycobacterium smegmatis*, polarization, TREM2, tuberculosis

## Abstract

**Background:**

This study aims to explore the association between Triggering Receptor Expressed on Myeloid Cells 2 (TREM2) expression and tuberculosis pathogenesis, with particular focus on its potential correlation with established macrophage polarization markers.

**Methods:**

The inflammatory pathology of lung tissues in mock-infected mice was evaluated using hematoxylin and eosin (H&E) staining. Additionally, the expression and localization of M1/M2 macrophage-specific markers and TREM2 were assessed via immunohistochemical (IHC) staining. The expression levels and localization of M1/M2 macrophage-associated cytokines, including IL-10 and IL-6, were measured in peripheral blood using enzyme-linked immunosorbent assay (ELISA) and quantitative real-time PCR (qRT-PCR). *Mycobacterium smegmatis* suspensions were co-cultured with THP-1-derived macrophages and RAW264.7 macrophages. Temporal changes in the expression of TREM2 and M1/M2 macrophage-specific markers were analyzed at different time points using qRT-PCR and Western blot.

**Results:**

I In the experimental group of mice, lung lesions exhibited widened alveolar septa, interstitial edema, and extensive infiltration of inflammatory cells and erythrocytes. TREM2 and inflammatory markers (iNOS and IL-10) showed significant positive expression in the infected lung tissues. Compared to the control group, the Mycobacterium-infected mouse model displayed significantly higher mRNA expression levels of TREM2, along with increased IL-10 and IL-6 production. Following co-culture with *Mycobacterium smegmatis*, both THP-1-derived macrophages and RAW264.7 cells exhibited upregulated TREM2 mRNA and protein expression. This upregulation was more closely associated with M2-type macrophage markers than with M1-type markers. Preferential TREM2 expression in M2 macrophages post-infection Notably, after *Mycobacterium smegmatis* infection, TREM2 expression was significantly more pronounced in M2-polarized macrophages compared to their M1 counterparts.

**Conclusion:**

TREM2 expression was significantly upregulated in lung tissues of tuberculosis-infected murine models, correlating strongly with increased expression of macrophage-derived cytokines. Notably, *in vitro* infection studies revealed that TREM2 upregulation followed a temporal pattern parallel to M2 macrophage marker expression. These findings suggest that TREM2 may be associated with M2 macrophage polarization and could potentially contribute to this phenotypic shift, a process that may play a critical role in the immunological mechanisms underlying tuberculosis pathogenesis.

## Introduce

Tuberculosis (TB), one of humanity’s oldest pandemics with origins that may trace back to prehistoric times, is associated with *Mycobacterium tuberculosis* (*Mtb*)—a pathogen that might have co-evolved with humans for approximately 70,000 years (Cosma et al., 2003; Monack et al., 2004). Despite centuries of coexistence, TB could continue to pose a significant global health burden, with more than 1.5 million annual deaths and 10 million new active cases worldwide potentially attributed to this disease (World Health Organization, 2020; Deutsch-Feldman et al., 2021). As an aerosol-transmitted pathogen, *Mtb* may primarily encounter alveolar macrophages upon entry into the host, where it might establish persistent infection. This interaction could trigger a complex array of host immune defenses, including inflammatory responses, phagocytosis, autophagy, and apoptosis, all of which might be aimed at eliminating the invading pathogen (Marino et al., 2015). Macrophages may not only act as the first line of defense against *Mtb* infection but also serve as the primary cellular niche for bacterial survival and replication (Refai et al., 2018). The plasticity and functional polarization of macrophages might play a pivotal role in determining host susceptibility versus resistance to TB. Deciphering the intricate host-pathogen interactions between *Mtb* and macrophages could reveal novel immunological targets, which may potentially facilitate the development of more effective vaccines and therapeutic interventions against TB.

Macrophages recognize *Mtb*-derived pathogen-associated molecular patterns (PAMPs) via various surface receptors, including Toll-like receptors (TLRs) and C-type lectin receptors (CLRs) (Schroder et al., 2004). This recognition triggers signaling cascades that promote the production of pro-inflammatory cytokines and activate anti-mycobacterial defense mechanisms, such as the generation of reactive nitrogen intermediates (RNI) and reactive oxygen species (ROS) (Fabri et al., 2011), as well as lysosomal fusion and autophagy (Bouchon et al., 2001). First identified in 2000, triggering receptor expressed on myeloid cells 2 (TREM2) is a transmembrane glycoprotein broadly expressed on immune cells, including macrophages, microglia, dendritic cells, and osteoclast precursors (Klesney-Tait et al., 2006). Functioning as a pathologically induced immune signaling hub, TREM2 binds to a variety of ligands—predominantly tissue damage markers (Wang et al., 2016; Gratuze et al., 2018)—to regulate immune responses during infection and inflammation. Under physiological conditions, TREM2 exerts tissue-specific activity; in contrast, during pathological states, it transforms into a critical immune signaling hub that coordinates the sensing and suppression of tissue damage. Structurally, TREM2 consists of an extracellular V-type immunoglobulin domain, a short extracellular region, a transmembrane helix, and a cytoplasmic tail that lacks intrinsic signaling motifs (Colonna, 2003). This receptor is involved in multiple biological processes, including cell survival, lipid metabolism, phagocytosis, and inflammatory regulation. Clinical evidence links TREM2 dysregulation to a range of chronic inflammatory and autoimmune disorders—most notably Alzheimer’s disease, malignancies, pulmonary fibrosis, and pneumonitis—characterized by increased TREM2 expression during disease progression. In microbial infections, TREM2 exhibits dual functionality: it not only detects bacterial and fungal pathogens (Hamerman et al., 2006) but also attenuates the secretion of pro-inflammatory cytokines (Hsieh et al., 2009), thereby acting as a negative regulator of immune-inflammatory responses. Its expression in RAW264.7 macrophages, bone marrow-derived macrophages, and alternatively activated macrophages highlights its central role in macrophage biology. Emerging research suggests that *Mtb* exploits TREM2-mediated anti-inflammatory mechanisms to evade immune surveillance (Dabla et al., 2022); however, the precise role of this receptor in tuberculosis pathogenesis remains to be further elucidated.

Emerging evidence underscores the crucial role of macrophage polarization in the host defense against *Mtb* infection (Wang et al., 2015). During *Mtb* infection, granuloma-associated macrophages undergo a characteristic foamy transformation, where M2 polarization significantly facilitates this process and promotes the intracellular persistence of bacteria (Wynn et al., 2013; Gordon et al., 2014). Interestingly, TREM2-mediated regulation of macrophage polarization has been documented in various disease contexts: in neuroinflammation, upregulated TREM2 drives the transition of microglia from an M1 to an M2 phenotype, thereby exerting neuroprotective effects (Wang et al., 2016); similarly, during viral respiratory infections, TREM2 enhances IL-3 production and promotes M2 polarization of lung macrophages (Zheng et al., 2017). Although these findings suggest that TREM2 may serve as a potential modulator of macrophage polarization, its specific role and the underlying molecular mechanisms during *Mtb* infection remain poorly elucidated. Building on our preliminary *in vivo* observations regarding the expression patterns of TREM2 and its correlation with macrophage polarization in tuberculosis, the present study employs comprehensive *in vitro* approaches to clarify the molecular mechanisms that govern TREM2-mediated macrophage polarization during Mtb infection. These investigations are anticipated to identify novel therapeutic targets and advance our understanding of tuberculosis (TB) pathogenesis.

## Methods

### Study participants

A total of 43 patients with active pulmonary tuberculosis were enrolled, including 23 males and 21 females, with a mean age of 49.51 ± 11.90 years. These patients were diagnosed at a tertiary Grade A hospital in Xinjiang between January 2023 and December 2024. Meanwhile, 37 healthy control subjects were recruited, comprising 21 males and 16 females, with a mean age of 40.27 ± 13.61 years. The healthy controls had no infectious diseases, with normal liver function, renal function, and chest CT findings; their sputum smears for acid-fast bacilli were negative, and they had no history of tuberculosis. Additionally, lung lesion tissues were collected from 40 pulmonary tuberculosis patients during treatment.

The diagnostic criteria were based on the WS 288–2017 Diagnosis of Pulmonary Tuberculosis issued by China in 2018. The inclusion and exclusion criteria were as follows: Patients were required to present typical clinical manifestations and signs of tuberculosis, such as low-grade fever, night sweats, and fatigue, and meet at least one of the following three conditions: 1) Sputum smear or culture positive for Mycobacterium tuberculosis; 2) Negative sputum results but typical active pulmonary tuberculosis on chest imaging; 3) Negative sputum results and pathologically confirmed tuberculous granulomatous lesions, or tuberculous lesions identified in pleural effusion or bronchoalveolar lavage fluid. Patients with other immune diseases, malignant tumors, pulmonary infections, or HIV infection were excluded.

### Cell culture, bacterial culture and infection

For experimental investigations, we employed standard biological materials including: (1) RAW264.7 cells (murine monocyte-macrophage leukemia cell line), (2) THP-1 cells (human monocyte-derived macrophage cell line), and (3) *Mycobacterium smegmatis* mc’155 strain. Cryopreserved cells were revived from -80 °C storage or liquid nitrogen and subsequently cultured in complete medium comprising Gibco basal medium supplemented with 10% Gibco fetal bovine serum under optimal growth conditions. THP-1 monocytes, maintained in suspension culture, were differentiated into adherent macrophages by treatment with 30μL phorbol 12-myristate 13-acetate (PMA) solution (diluted 1:10 in RPMI-1640 medium) per 2 mL cell suspension in each well. Following 24-hour incubation at 37 °C with 5%CO_2_ to establish monolayer adherence, polarized macrophage subsets were generated through cytokine stimulation: M1 polarization was induced with 100ng/mL lipopolysaccharide (LPS) plus 20ng/mL interferon-γ (IFN-γ), while M2 polarization was achieved using 20ng/mL interleukin-4 (IL-4) and 20 ng/mL interleukin-13 (IL-13).

The frozen stock of *Mycobacterium smegmatis* was thawed in a 37 °C water bath, and a bacterial suspension was prepared by transferring a loopful of culture using a sterile inoculating loop onto 7H10 solid medium in a 10cm Petri dish. Using the three-zone streaking method, the inoculated plate was incubated inverted at 37 °C with 5% CO_2_. After 24–36 hours of incubation, distinct milky-white colonies with rough, moist morphology became visible. Well-isolated single colonies were then transferred to liquid medium and cultured under constant agitation (140-160rpm) at 37 °C with 5%CO_2_ until reaching optimal growth density for subsequent cell infection experiments.

Macrophages were distributed in a six-well plate at a cell density of 4*×*10^5^ cells/ml, and following complete adhesion to the wall, the cells were placed in a 37°C, 5%CO^2^ cell culture incubator for a 24h period. The complete medium was then discarded, and 100μL of *Mycobacterium smegmatis* suspension was diluted 10-fold with 7H9 liquid medium to a final volume of 1 mL, which was added to each well. The multiplicity of infection (MOI) was set at 10:1 (bacteria: cells). It should be noted that the CFU value was estimated based on optical density (OD_600_) measurements, as parallel plate counting was not performed for each experiment. The control group received 1ml of PBS and 1ml of cell culture medium without double antibiotics. Following a two-hour incubation period in an incubator maintained at 37 °C with 5%CO^2^, the liquid in the six-well plate was gently discarded, and the plate was washed twice with basic medium. The plate was then replaced with complete cell culture medium containing double antibiotic, and placed in the cell culture incubator at 37 °C with 5%CO^2^ for further incubation. The cell culture medium or cells were collected at the designated time points for subsequent measurement. All infection experiments were performed with at least three independent biological replicates (n ≥ 3).

### RNA and protein extraction

Protein and RNA extraction were performed using standardized protocols. For protein isolation, cells from 6-well plates were harvested in microcentrifuge tubes and lysed using RIPA buffer supplemented with PMSF (1:100 ratio). Following centrifugation at 12,000 × g for 15 minutes at 4 °C, the protein-containing supernatant was carefully transferred to fresh tubes and stored at -80 °C. For RNA extraction, cells were directly lysed in plates using TRIzol reagent, followed by transfer to microcentrifuge tubes. After adding chloroform (200μL per 1mL TRIzol) and phase separation by centrifugation, the aqueous phase was mixed with an equal volume of isopropanol to precipitate RNA. The RNA pellets obtained by centrifugation were washed with 75% ethanol and resuspended in RNase-free water. All centrifugation steps were performed at 4 °C to maintain sample integrity.

### Detection of mRNA expression levels

RNA quality assessment and cDNA synthesis were performed according to standardized molecular biology protocols. RNA concentration and purity were quantified spectrophotometrically, with samples meeting stringent quality control criteria (≥80 ng/μL concentration and A260/A280 ratio of 2.0 ± 0.2) being selected for downstream applications. Reverse transcription was carried out using the Takara Prime Script RT reagent kit following manufacturer’s specifications, with reactions performed in a thermal cycler under optimized cycling conditions. For quantitative real-time PCR (qRT-PCR), reactions were established using the Takara TB Green Premix Ex Taq II kit (Cat. No. RR820A). All procedures were conducted under RNase-free conditions to ensure experimental reproducibility. All primer sequences used in this study are listed in **Table 1**.

**Table 1:** Primer sequence.

Gene (human,mouse)	Primer (5’→3’)
TREM2	Forward:CCAGCCTGCATACTTGCCA
	Reverse:GGCAGAGTAGTCTCTTGCCAG
IL-6	Forward:ACTCACCTCTTCAGAACGAATTG
	Reverse:CCATCTTTGGAAGGTTCAGGTTG
TNF-α	Forward:GAGGCCAAGCCCTGGTATG
	Reverse:CGGGCCGATTGATCTCAGC
IL-1β	Forward:TTCGACACATGGGATAACGAGG
	Reverse:TTTTTGCTGTGAGTCCCGGAG
IL-10	Forward:GACTTTAAGGGTTACCTGGGTTG
	Reverse:TCACATGCGCCTTGATGTCTG
TREM2	Forward:CTGGAACCGTCACCATCACTC
	Reverse:CGAAACTCGATGACTCCTCGG
IL-6	Forward:CCAAGAGGTGAGTGCTTCCC
	Reverse:CTGTTGTTCAGACTCTCTCCCT
IL-10	Forward:GCTCTTACTGACTGGCATGAG
	Reverse:CGCAGCTCTAGGAGCATGTG
TNF-a	Forward:CCCTCACACTCAGATCATCTTCT
	Reverse:GCTACGACGTGGGCTACAG
GAPDH	Forward:CATCCACTGGTGCTGCCAAGGCTGT
	Reverse:ACA ACCTGGTCCTCAGTGTAGCCCA

### Elisa

The concentrations of IL-10 and IL-6 in plasma samples from both experimental and control groups were quantitatively determined using a commercial ELISA kit (Jianglai Biotechnology, Shanghai, China) following the manufacturer’s standardized protocol.

### HE, IHC staining, Western blot

Histological and immunohistochemical analyses were performed using standardized protocols. For HE staining, tissue sections were sequentially treated with: (1) Mayer’s hematoxylin for 3–5 minutes, (2) 1% acid alcohol for differentiation, (3) eosin Y counterstain for 1 minute, and (4) neutral balsam mounting medium. IHC staining involved antigen retrieval using 10 mM sodium citrate buffer (pH 6.0) at 95 °C for 15 minutes, followed by blocking with 10% normal goat serum for 1h at room temperature. Primary antibody incubation was performed overnight at 4 °C at optimized concentrations, followed by appropriate HRP-conjugated secondary antibody incubation at 37 °C for 30 minutes. Color development was achieved using DAB substrate, with hematoxylin counterstaining for nuclear visualization. For IHC staining, five random high-power fields (×400) per section were scored independently by two blinded observers using a 0–3 scale (0: none, 1: weak, 2: moderate, 3: strong). The mean score was used for analysis.

The following antibodies were used in this study: Primary antibodies used were: anti-TREM2 (Bioss, bs-2723R; 1:1000 for WB, 1:200 for IHC), anti-iNOS (Abcam, ab178945; 1:1000 for WB, 1:100 for IHC), anti-Arg-1 (Cell Signaling Technology, 93668S; 1:1000 for WB), and anti-GAPDH (Proteintech, 60004-1-Ig; 1:5000 for WB). HRP-conjugated goat anti-rabbit and anti-mouse secondary antibodies (Abcam, ab205718 and ab205719) were used at 1:5000 for WB. Western blot densitometry: Band intensities were quantified using ImageJ software. The relative expression level of each target protein was obtained by normalization its net intensity to that of GAPDH (internal control). Each band was measured three times, and the mean value was used for analysis.

### Establishment of a mouse model of *Mycobacterium smegmatis* simulated infection

Thirty female BALB/c mice of 6–8 weeks of age and weighing 18-24g were selected from the Experimental Animal Centre of Xinjiang Medical University. Mice were randomly assigned to the control and experimental groups using a random number table, with 15 mice per group. Thirty female BALB/c mice (6–8 weeks old, 18–24 g) were obtained from the Experimental Animal Center of Xinjiang Medical University and randomly allocated into two groups (n=15 per group). For infection preparation, *Mycobacterium smegmatis* colonies were homogenized in sterile saline containing 0.05% Tween-80 to create a uniform bacterial suspension. Experimental group mice received intranasal inoculation with 5×10^6^ CFU/day of M. smegmatis, while control animals received equivalent volumes of sterile PBS. Following a 4-week infection period, mice were euthanized for tissue collection. Gross pathological examination of thoracic and abdominal cavities was performed prior to tissue harvesting. Lung tissues were aseptically dissected, with portions either: (1) fixed in 4% paraformaldehyde for 24h (followed by PBS washing and 75% ethanol storage) for HE and IHC analyses, or (2) immediately snap-frozen in liquid nitrogen and stored at -80 °C for subsequent RNA extraction and qRT-PCR studies. All procedures were conducted under biosafety level 2 containment conditions.

### Statistical analysis

Statistical analyses were performed using GraphPad Prism (v8.0.1), SPSS (v26.0), ImageJ, and RStudio (v4.0.3) software packages. All experiments were independently repeated at least three times, and the data are presented as the mean ± standard deviation (SD). Normality of the data was evaluated using the Shapiro-Wilk test. For comparisons between two groups, an unpaired two-tailed Student’s t-test was employed for normally distributed data, whereas the Wilcoxon rank-sum test was utilized for non-normally distributed data. For comparisons among multiple groups (including those involving time courses and different polarization states), one-way or two-way analysis of variance (ANOVA) was performed, followed by *post hoc* multiple comparison tests with Tukey’s or Šidák’s correction. Prior to statistical analysis, densitometric values from Western blot analyses were normalized to the corresponding loading control. For *in vitro* experiments, data represent results from at least three independent biological replicates (n≥3), with each biological replicate measured in triplicate as technical replicates. For the animal study, 15 mice were assigned to each group (n=15), and tissues were collected from 3–5 mice per time point. A p-value < 0.05 was considered statistically significant.

### Ethical approval explanation

All human specimen-related procedures in this study were approved by the Ethics Committee of Xinjiang Medical University Affiliated Tumor Hospital (Approval No.: S-2024122). Animal experimental protocols were reviewed and approved by the Animal Ethics Committee of Xinjiang Medical University (Approval No.: IACUC-20230523-27).

## Result

### Histopathological manifestations of the lungs of mice simulating *Mycobacterium smegmatis* infection

**Figure 1:**
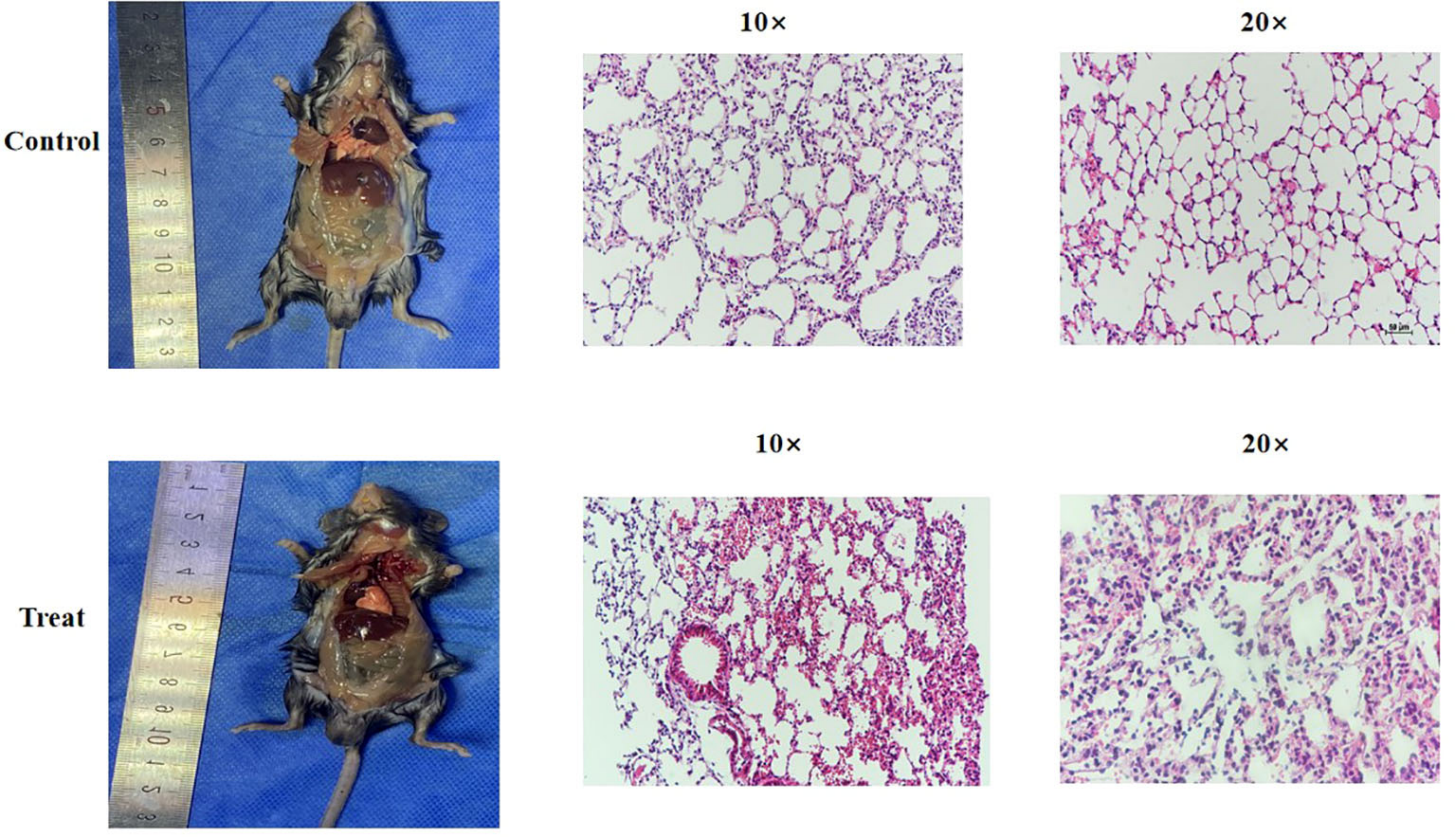
Histopathologic staining results of lungs in a mouse model of tuberculosis.

**Figure 2:**
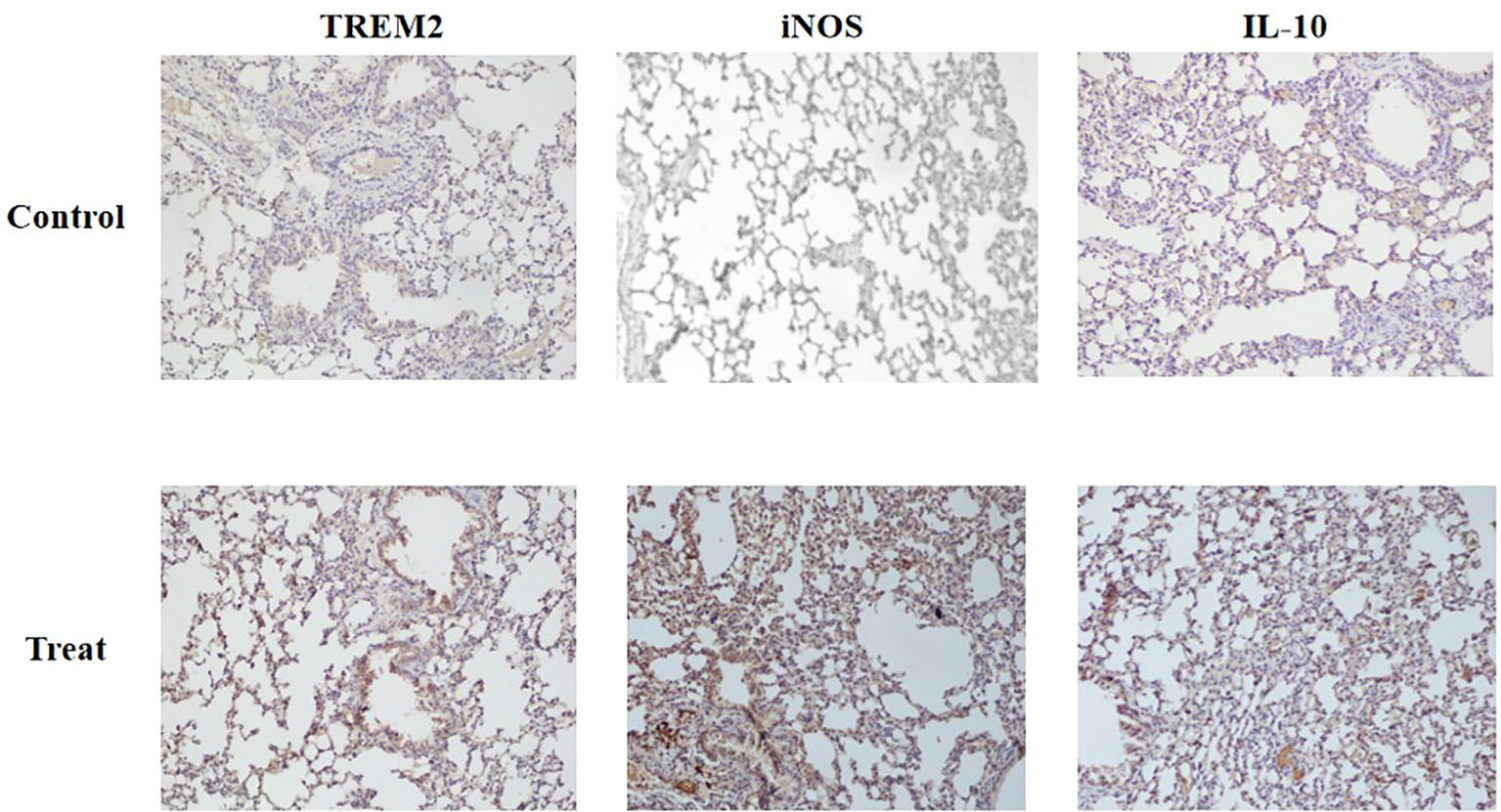
Expression of TREM2 and inflammatory cytokines in lung tissues of mice infected with Mycobacterium smegmatis.

*Mycobacterium smegmatis*, a rapidly growing non-tuberculous mycobacterium sharing genomic homology with *Mtb*, demonstrates avirulence in rodent models at low inoculation doses. HE (**Figure 1**) revealed distinct morphological differences between experimental and control groups: control lung tissues maintained normal pulmonary architecture with intact alveoli and minimal inflammatory cell infiltration, whereas infected specimens exhibited significant pathological alterations including alveolar septal thickening, interstitial edema, and extensive infiltration of both inflammatory cells and erythrocytes. IHC analysis (**Figure 2**) demonstrated markedly elevated expression of TREM2 and inflammatory markers (iNOS and IL-10) in infected tissues compared to controls, with characteristic tan-colored immunoreactive deposits localized to granulomatous regions. Semi-quantitative scoring confirmed statistically significant between-group differences in both staining intensity and distribution patterns.

### Study of TREM2 and inflammatory cytokine expression in lung tissues of *Mycobacterium smegmatis*-infected mice

**Figure 3:**
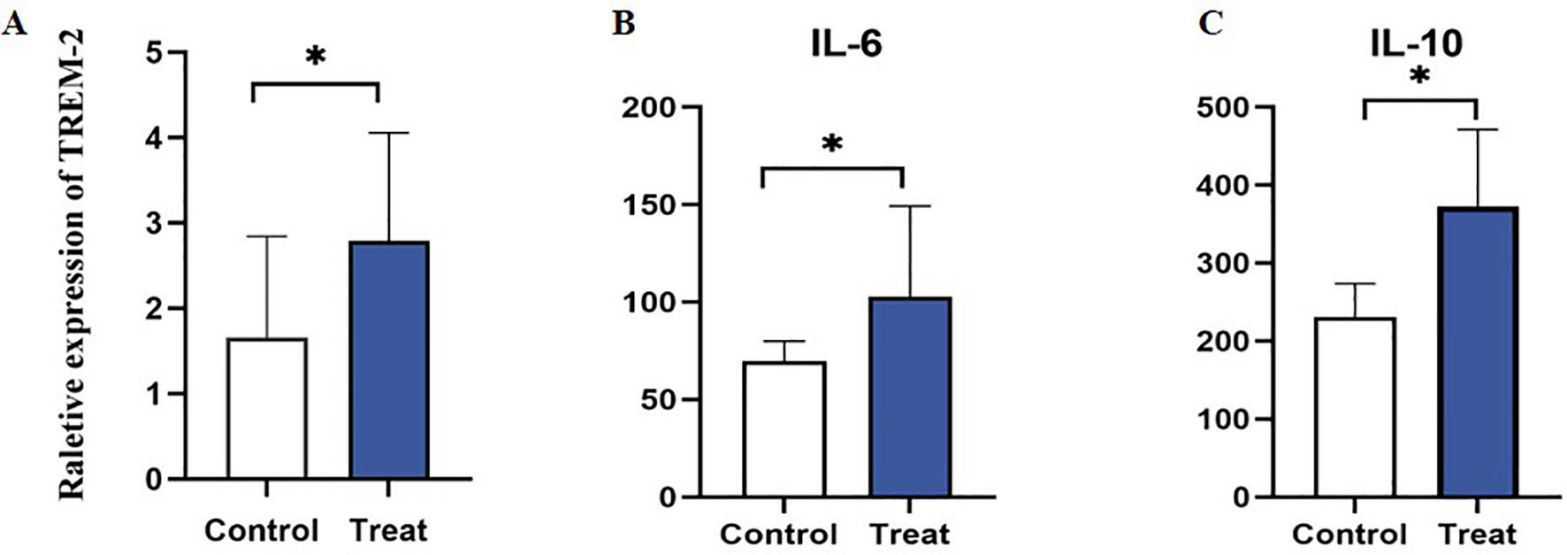
Results of qRT-PCR and ELISA in a mouse model of tuberculosis. **(A)** Relative expression levels of TREM2 mRNA in infected and control mice **(B)** Comparison of IL-6 expression levels in blood of infected and control mice **(C)** Comparison of IL-10 expression levels in blood of infected and control mice, *p<0.05.

The expression levels of TREM2 (**Figure 3**) and the macrophage-associated cytokines IL-6 (**Figure 3**) and IL-10 (**Figure 3**) in the *Mycobacterium smegmatis*-infected mouse model were measured by qRT-PCR and ELISA. The results are presented in **Figure 3** and **Table 2**. The expression of TREM2 mRNA was found to be significantly up-regulated in the lung tissues of infected mice, and the expression of IL-6 and IL-10 in the serum of infected mice was significantly increased, with the expression levels of IL-10 being 265.9 ± 32.7 pg/ml and 109.2 ± 15.68 pg/ml, respectively. The difference between these two levels was found to be statistically significant (*P* < 0.05).

**Table 2:** Relative expression levels of TREM2 mRNA , IL-6,IL-10 in lung tissues of mouse infection models (x±s).

Cytokine	Control group	Treat group	*P*
TREM2 mRNA	1.75±1.56	2.89±2.55	<0.05
IL-6(pg/ml)	72.6±8.56	109.2±15.68	<0.05
IL-10(pg/ml)	212.6±10.65	265.9±32.7	<0.05

### Effect of co-culture of *Mycobacterium smegmatis* with macrophages on macrophage polarization

**Figure 4:**
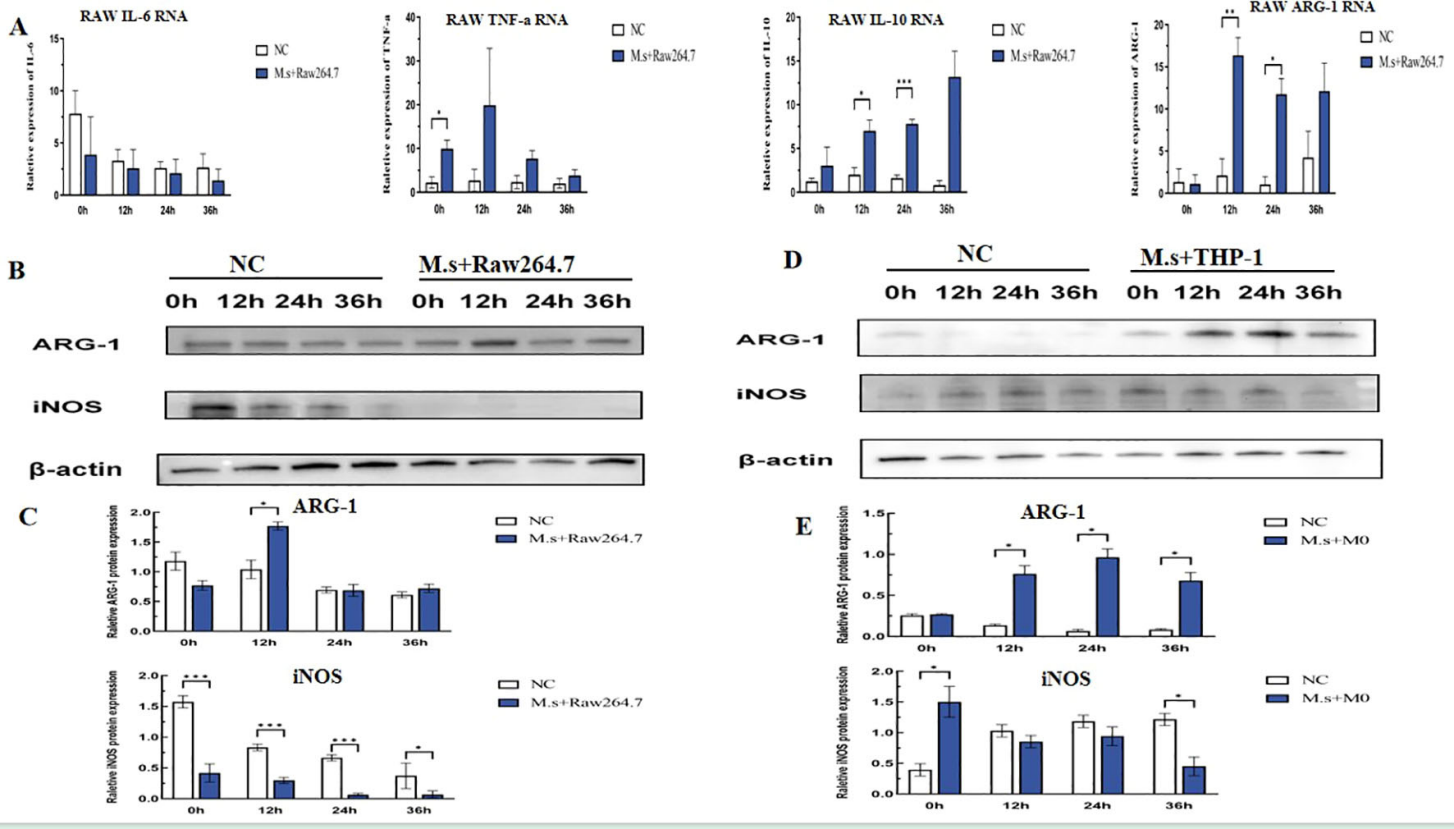
Expression of various correlates at different detection time points during Mycobacterium smegmatis infection of RAW264.7 and THP-1-derived macrophages. **(A)** mRNA expression levels of each indicator after Mycobacterium pubescens infection of RAW264.7 macrophages at different time points **(B)** Protein expression levels of Arg-1,iNOS after Mycobacterium pubescens infection of RAW264.7 macrophages **(C)** Statistical graphs of protein expression levels of Arg-1,iNOS **(D)** Arg-1,iNOS protein expression levels of Mycobacterium pubescens infection of THP-1 macrophages **(E)** Statistical graphs of protein expression levels of Arg-1,iNOS; *p<0.05,**p<0.01; *p<0.05,**p<0.01. -1,iNOS protein expression level **(E)** Statistical of Arg-1,iNOS protein expression level. *p<0.05, **p<0.01, ***p<0.001, (n=3).

During tuberculosis pathogenesis, macrophages undergo distinct polarization toward the M2-type, which mediates immunosuppression through enhanced production of anti-inflammatory cytokines. This phenotypic shift facilitates immune evasion by *Mtb*, enabling persistent infection and disease progression qRT-PCR experiments have demonstrated that the mRNA expression level of TNF-α in the experimental group was elevated at 12 h, followed by a gradual downward modulation, and that the mRNA expression level of IL-6 showed an overall decreasing trend with the prolongation of the time of infection (**Figure 4**, **Table 3**). Conversely, the mRNA expression levels of M2 macrophage markers Arg-1 and IL-10 in the experimental group were higher than those in the control group at 12 and 24 h (*P* < 0.01), and the differences were statistically significant (**Figure 4**, **Table 4**).

**Table 3:** Relative expression levels of IL-6,TNF-a mRNA after Mycobacterium smegmatis infection of RAW264.7 macrophages (x±s).

Time	Group	IL-6 mRNA expression	t	*P*	TNF-a mRNA expression	t	*P*
0h	Control	7.84±2.18	1.62	0.78	2.26±1.29	5.62	0.03
	Treat	3.89±3.61			9.94±1.98		
12h	Control	3.31±1.08	0.59	>0.99	2.73±2.51	2.23	0.58
	Treat	2.59±1.8			19.87±13.03		
24h	Control	2.61±0.61	0.58	>0.99	2.35±1.52	3.91	0.07
	Treat	2.12±1.33			7.71±1.83		
36h	Control	2.65±1.32	1.25	>0.99	2.05±1.1	1.80	0.59
	Treat	1.4±1.11			3.85±1.33		

**Table 4:** Relative expression levels of IL-10,Arg-1 mRNA after Mycobacterium smegmatis infection of RAW264.7 macrophages (x±s).

Time	Group	IL-10 mRNA expression	t	*P*	Arg-1 mRNA expression	t	*P*
0h	Control	1.25±0.36	1.45	>0.99	1.34±1.57	0.19	>0.99
	Treat	3.05±2.12			1.13±1.07		
12h	Control	2.01±0.79	5.88	0.03	2.1±2	8.53	0.004
	Treat	7.02±1.24			16.36±2.09		
24h	Control	1.63±0.33	18.13	<0.001	1.06±0.92	26.02	<0.001
	Treat	7.83±0.49			19.43±0.81		
36h	Control	0.81±0.53	7.21	0.06	4.26±3.09	5.35	0.08
	Treat	13.2±2.93			14.47±1.17		

In order to verify the experimental results obtained above, *Mycobacterium smegmatis* was co-cultured with RAW264.7 macrophages and M0-type THP-1 macrophages for 0, 12, 24 and36h. For this investigation, we selected iNOS and Arg-1 as canonical biomarkers representing opposing macrophage polarization states (M1 and M2 phenotypes, respectively). The findings of the WB experiments demonstrated that following the co-culture of *Mycobacterium smegmatis* with RAW264.7 macrophages, the expression levels of iNOS protein in the experimental group were diminished relative to those in the control group with the prolongation of the infection time (*P* < 0.01), and the discrepancies were all statistically significant (**Figure 4D**, **Table 5**). Following the co-culture of *Mycobacterium smegmatis* and M0 type THP-1 macrophages (**Table 6**), the protein expression level of iNOS in the experimental group exhibited an initial increase at 0 hours, followed by a gradual decrease, resulting in a level that was lower than that observed in the control group at 36 h. This difference was found to be statistically significant. The protein expression level of Arg-1, a marker for M2 macrophages, increased in the RAW264.7 experimental group at 12 hours (*P* < 0.05) and exhibited a tendency to be comparatively higher than that in the control group (**Figure 4C**). In the THP-1 experimental group, the protein expression level of Arg-1 was higher than that of the control group at the middle and end stages of infection (*P* < 0.05), and the differences were statistically significant. Our findings demonstrate a significant polarization shift toward the M2 macrophage phenotype during *Mycobacterium smegmatis* infection (*P* < 0.05).

**Table 5:** Protein expression levels of Arg-1,iNOS after Mycobacterium smegmatis infection of RAW264.7 macrophages respectively (x±s).

Time	Group	RAW264.7 iNOS protein expression	t	*P*	RAW264.7 Arg-1 protein expression	t	*P*
0h	Control	1.57±0.1	13.57	<0.001	1.18±0.15	4.10	0.10
	Treat	0.42±0.15			0.77±0.08		
12h	Control	0.83±0.06	6.29	<0.001	1.04±0.16	7.41	0.03
	Treat	0.3±0.05			1.77±0.07		
24h	Control	0.66±0.05	7.03	<0.001	0.69±0.05	0.12	>0.99
	Treat	0.07±0.02			0.69±0.1		
36h	Control	0.37±0.21	3.53	0.01	0.61±0.05	2.20	0.40
	Treat	0.07±0.06			0.72±0.07		

**Table 6:** Relative expression levels of TREM2 mRNA, protein after Mycobacterium smegmatis infection with RAW264.7 (x±s).

Time	Group	RAW264.7 TREM2 mRNA expression	t	*P*	RAW264.7 TREM2 protein expression	t	*P*
0h	Control	2.14±0.99	2.34	0.33	0.45±0.06	6.317	0.05
	Treat	1.85±1.97			1.07±0.16		
12h	Control	0.90±0.76	6.66	0.02	0.54±0.07	4.604	0.04
	Treat	1.95±2.32			0.81±0.07		
24h	Control	0.49±0.88	5.99	0.04	0.54±0.05	7.152	0.01
	Treat	1.75±0.53			0.92±0.08		
36h	Control	0.66±0.43	0.41	>0.99	1.14±0.15	6.605	0.04
	Treat	0.49±0.39			0.5±0.07		

### Expression of TREM2 in macrophages after *Mycobacterium smegmatis* infection

**Figure 5:**
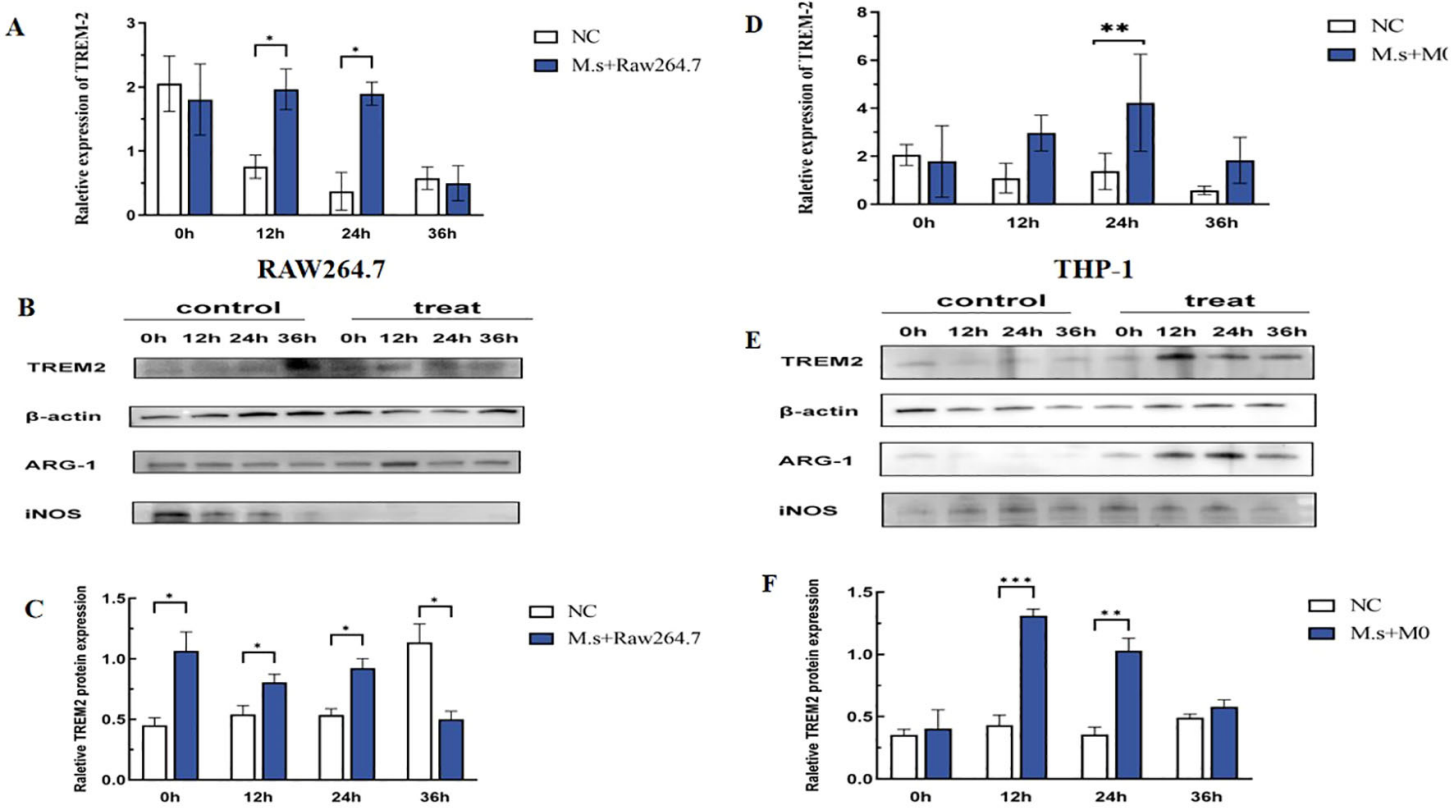
Expression of TREM2 at different detection time points in Mycobacterium smegmatis infected RAW264.7 and THP-1-derived macrophages. **(A)** RT-qPCR results of TREM2 after Mycobacterium pubescens infection of RAW264.7 macrophages **(B)** Protein expression level of TREM2 after Mycobacterium pubescens infection of RAW264.7 macrophages **(C)** Statistical graph of TREM2 protein expression level **(D)** RT-qPCR results of TREM2 after Mycobacterium pubescens infection of THP-1 macrophages **(E)** Protein expression level of TREM2 after Mycobacterium pubescens infection of THP-1 macrophages **(F)** Statistical graph of TREM2 protein expression level. *p<0.05, **p<0.01, ***p<0.001, (n=3).

The mRNA and protein levels of TREM2 in *Mycobacterium smegmatis* co-cultured with RAW264.7 macrophages and M0-type THP-1 macrophages, respectively, were detected by qRT-PCR and WB (**Figure 5**, **Table 6**). When *Mycobacterium smegmatis* was co-cultured with RAW264.7 macrophages for 0, 12, 24 and 36h, the qRT-PCR results showed that the mRNA expression level of TREM2 in the experimental group was up-regulated at 12 and 24 h, and had a slight decreasing tendency at 36 h (**Figure 5**). The protein expression was detected by Western Blot, and compared with the control group, the expression level of TREM2 protein was up-regulated at 0h, 12h and 24h, and the difference was statistically significant (**Figure 5B**).

When *Mycobacterium smegmatis* was co-cultured with THP-1 macrophages for 0,12,24,36h, the mRNA expression level of TREM2 in the experimental group was up-regulated at 24h (**Figure 5**). Conversely, the protein expression level was elevated at 12h and 24h in comparison to the control group (**Figure 5F**, **Table 7**). The results indicated that following co-culture with *Mycobacterium smegmatis* and macrophages, TREM2 expression was elevated in the experimental group, reaching statistical significance compared to the control group after 12 and 24h of co-culture. Furthermore, the up-regulation of TREM2 protein expression was more pronounced in THP-1 than in RAW264.7.

**Table 7:** Relative expression levels of TREM2 mRNA, protein after Mycobacterium smegmatis infection with THP-1 (x±s).

Time	Group	THP-1 TREM2 mRNA expression	t	*P*	THP-1 TREM2 protein expression	t	*P*
0h	Control	2.05±0.43	0.29	>0.99	0.35±0.05	0.53	>0.99
	Treat	1.79±1.49			0.4±0.15		
12h	Control	1.09±0.62	3.10	0.19	0.43±0.08	15.74	<0.001
	Treat	2.97±0.74			1.31±0.05		
24h	Control	1.37±0.76	6.66	0.04	0.36±0.06	9.93	0.005
	Treat	4.23±2.02			1.03±0.1		
36h	Control	0.58±0.18	2.23	0.59	0.49±0.03	2.29	0.42
	Treat	1.83±0.96			0.58±0.06		

To quantitatively assess the relationship between TREM2 and M2 polarization, Pearson correlation analysis was performed using data from three independent experiments across the four time points. In THP-1 cells, TREM2 expression showed a significant positive correlation with the M2 marker Arg-1 at both mRNA (r=0.66, *p* = 0.019) and protein levels (r=0.71, *p* = 0.009). In contrast, no significant correlation was observed in RAW264.7 cells (mRNA: r=0.018, *p* = 0.956; protein: r=-0.016, *p* = 0.96).The analysis results showed that the expression levels of TREM2 and M2 type macrophage markers Arg-1, IL-10 were higher than those of the control group, and the expression levels were significantly up-regulated in the experimental group after 12 and 24h of infection, and the differences were statistically significant. These results suggest that the expression of TREM2 is positively associated with M2 macrophage polarization in a cell-type-specific manner, with a robust correlation evident in human THP-1 macrophages.

### Induced differentiation and characterization of THP-1 cells

**Figure 6:**
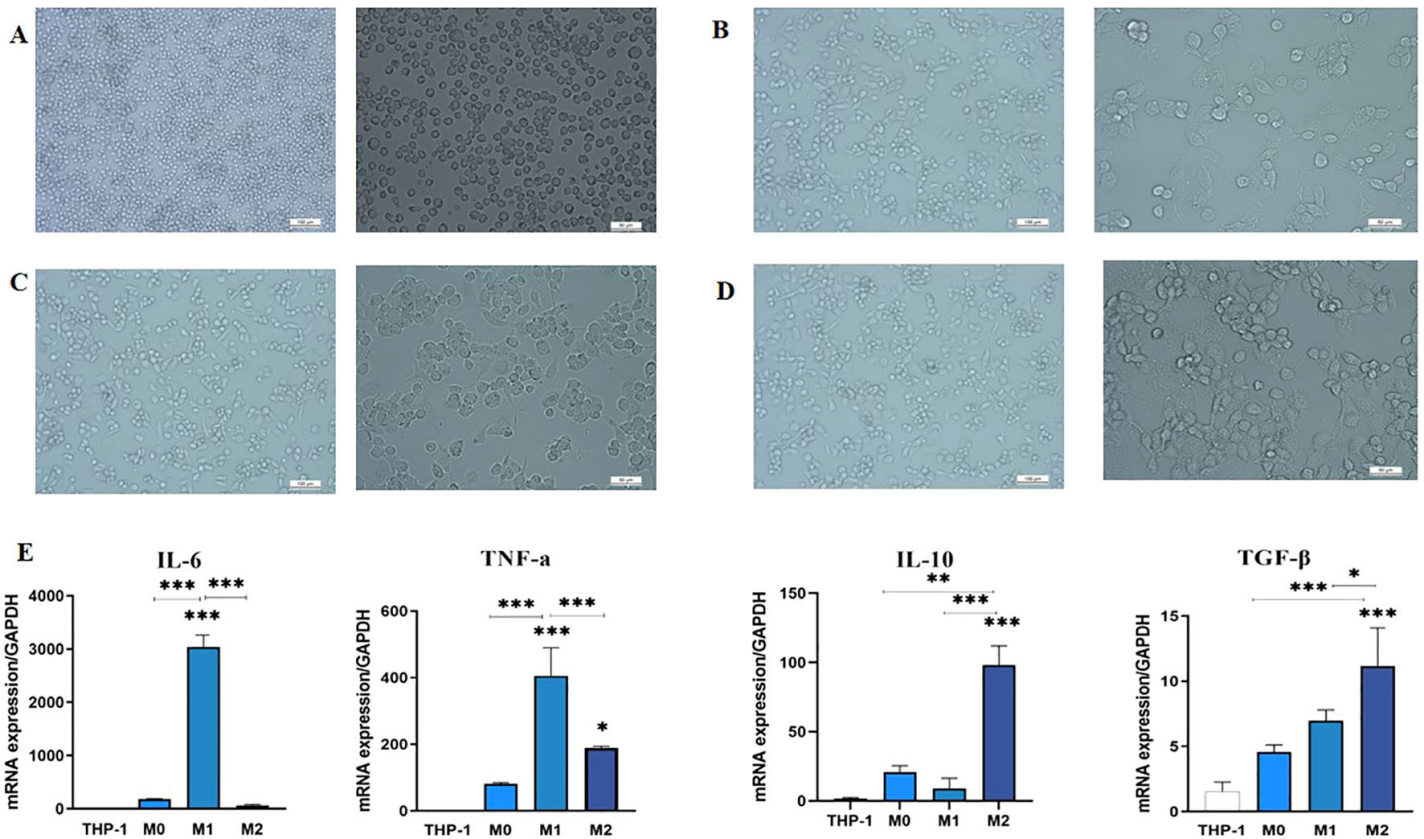
Morphological changes in induced differentiation of THP-1 cells and detection of mRNA of specific markers. **(A)** THP-1 cells (×100, ×200) **(B)** M0 macrophages (×100, ×200) **(C)** M1-type macrophages (×100, ×200) **(D)** M2-type macrophages (×100, ×200) **(E)** Relative mRNA expression levels of TNF-α,IL-6, IL-10, TGF-β. *p<0.05, **p <0.01, ***p <0.001, (n=3).

M0 could be observed after 12 h of induction of wall-adherent THP-1 cells (**Figure 6A**). The models of M1 (**Figure 6**) and M2 type macrophages (**Figure 6**) were successfully established *in vitro* using the cytokine induction technique, and the expression levels of TNF-α, IL-6, markers of M1 type macrophages, and TGF-β Arg-1, a marker of M2 type macrophages, were detected by qRT-PCR (**Figure 6**, **Table 8**). The results showed that the expression levels of TNF-α and IL-6 in M1-type macrophages were higher than those in M0- and M2-type macrophages, and the differences were statistically significant, suggesting that the *in vitro* M1-type macrophage induction model was successfully established. Similarly, the expression levels of TGF-β and Arg-1 in M2-type macrophages were higher than those in M0 and M1-type macrophages, indicating that the *in vitro* M2-type macrophage induction model had been successfully constructed.

**Table 8:** Detection of specific marker mRNA after THP-1-induced differentiation (x±s).

分组	IL-6	TNF-A	TGF-B	IL-10
THP-1	2.07±1.15	1.24±0.43	1.53±0.74	1.53±0.98
M0	183.78±6.67	81.16±4.15	4.57±0.54	13.21±9.56
M1	3043.22±219	405.07±84.64	6.98±0.82	15.67±5.89
M2	64.09±18.5	189.67±4.45	11.16±2.94	89.14±24.23

### TREM2 expression is associated with M2-type macrophages

**Figure 7:**
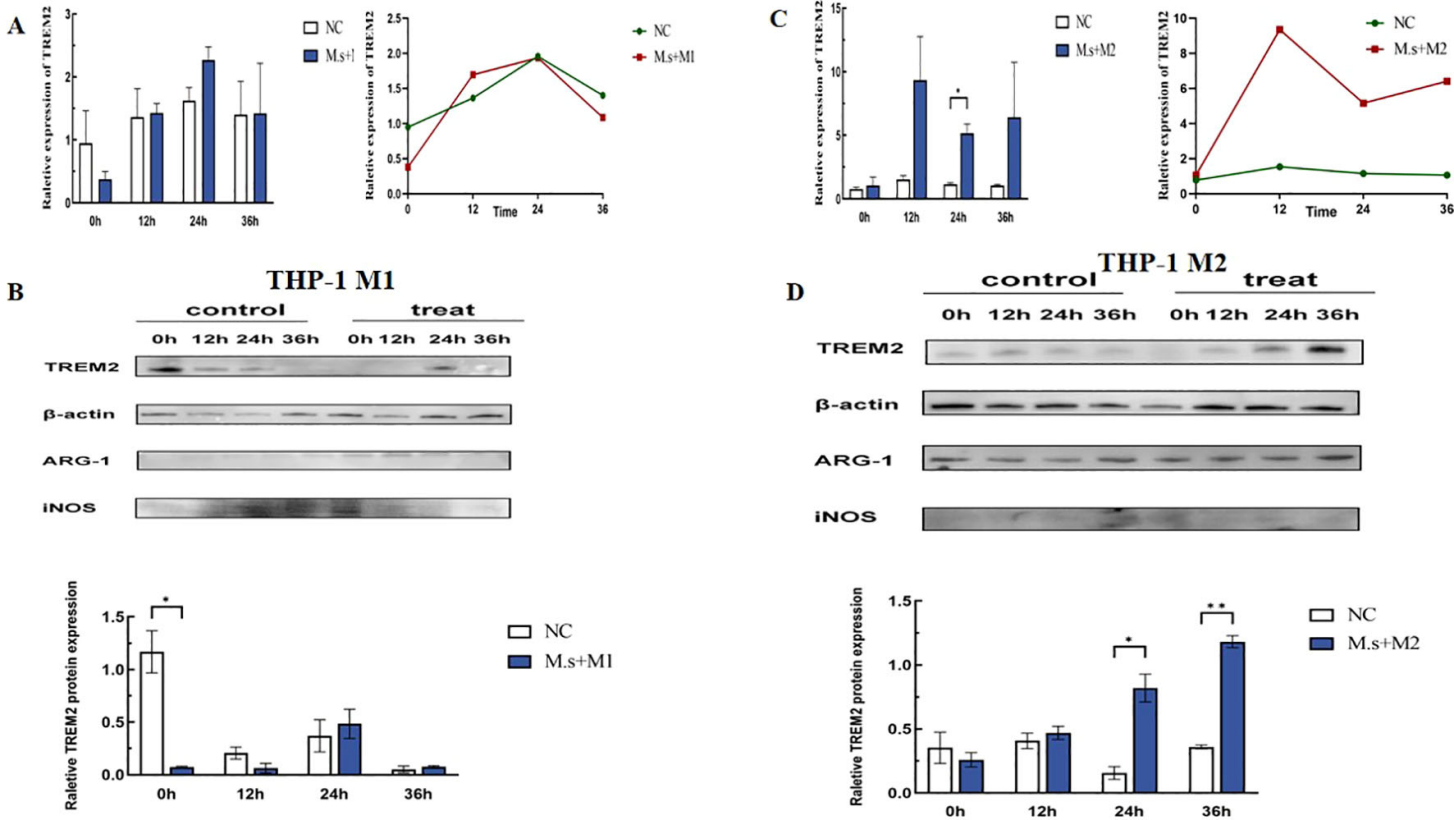
Expression of TREM2 at different detection time points during Mycobacterium smegmatis infection of M1/M2 sexual macrophages. **(A)** RT-qPCR results of TREM2 after Mycobacterium pubescens infection of M1-type macrophages **(B)** Protein expression level and statistics of TREM2 after Mycobacterium pubescens infection of M1-type macrophages **(C)** RT-qPCR results of TREM2 after Mycobacterium pubescens infection of M2-type macrophages **(D)** Protein of TREM2 after Mycobacterium pubescens infection of M2-type macrophages expression levels and statistical plots. *p<0.05, **p<0.01.

*Mycobacterium smegmatis* was co-cultured with M1/M2 macrophages for 0,12,24 and 36h, and THP-1 cells were collected for mRNA and protein levels of TREM2. PCR results showed that there was no statistically significant difference in the expression level of TREM2 between *Mycobacterium smegmatis* and M1 macrophages after co-culturing (*P*>0.05) (**Figure 7**, **Table 9**), while TREM2 expression level was significantly up-regulated (*P* < 0.05) at 24 and 36h after co-culturing with M2 macrophages and continued to be expressed at high levels (**Figure 7**, **Table 9**). M2-type macrophages co-culture, the expression level of TREM2 in the experimental group was significantly up-regulated at 24, 36h (*P* < 0.05), and continued to maintain a high level of expression (**Figure 7**, **Table 10**). WB results showed that the protein expression of TREM2 was more pronounced in M2-type macrophages (*P* < 0.01), and with the extension of the infection time overall showed an upward trend (**Figure 7B**).

**Table 9:** Relative mRNA and protein expression levels of TREM2 after Mycobacterium smegmatis infection of M1 macrophages (x±s).

Time	Group	M1 TREM2 mRNA expression	t	*P*	M1 TREM2 protein expression	t	*P*
0h	Control	0.95±0.51	1.89	0.75	1.17±0.2	9.47	0.04
	Treat	0.38±0.12			0.07±0.01		
12h	Control	1.36±0.45	0.23	>0.99	0.21±0.06	3.40	0.12
	Treat	1.43±0.15			0.06±0.05		
24h	Control	1.62±0.21	3.79	0.08	0.37±0.15	0.95	>0.99
	Treat	2.27±0.21			0.48±0.14		
36h	Control	1.4±0.53	0.04	>0.99	0.05±0.03	1.43	>0.99
	Treat	1.42±0.8			0.08±0.01		

**Table 10:** Relative mRNA and protein expression levels of TREM2 after Mycobacterium smegmatis infection of M2 macrophages (x±s).

Time	Group	M2 TREM2 mRNA expression	t	*P*	M2 TREM2 protein expression	t	*P*
0h	Control	0.78±0.15	0.72	>0.99	0.35±0.12	1.20	>0.99
	Treat	1.06±0.66			0.26±0.06		
12h	Control	1.53±0.3	3.94	0.23	0.41±0.06	1.35	>0.99
	Treat	9.35±3.42			0.47±0.05		
24h	Control	1.16±0.11	9.62	0.04	0.16±0.05	9.56	0.01
	Treat	5.17±0.71			0.82±0.11		
36h	Control	1.07±0.08	2.14	0.66	0.36±0.02	28.77	0.002
	Treat	6.41±4.33			1.18±0.05		

## Discussion

The ability of *Mtb* to survive and cause disease is closely linked to its ability to evade immune protection mechanisms (Seno et al., 2009; Roca et al., 2019). TREM2 is considered to be a novel immunomodulatory receptor, It has been demonstrated that TREM2 regulates the secretion of cytokines or extracellular vesicles by macrophages in sepsis, toxemia, inflammatory bowel disease and other pathophysiological processes related to inflammatory response (Correale et al., 2013; Genoula et al., 2020). However, there is a paucity of literature on the subject of TREM2 in the context of tuberculosis.

In the context of inflammatory lung diseases, TREM2 expression has been observed in various lung cell types, including alveolar macrophages and fibroblasts (Castillo et al., 2017; Kong et al., 2020). Wu et al. reported an increase in TREM2 expression on the surface of lung macrophages and epithelial cells in mice during the initial stage of viral infection. This increase led to the inhibition of the apoptosis of these cells, which would have otherwise occurred during the acute phase of the diseases (Wu et al., 2015). Mycobacterium smegmatis is widely used as a TB model organism due to its fast growth, safety, and shared virulence genes with M. tuberculosis (Wang et al., 2020). In this study, an infected mouse model was constructed by using *Mycobacterium smegmatis* to mimic *Mtb*. Immunohistochemistry showed that TREM-2 was mainly expressed in the cell cytosol and cytoplasm, and a significant positive expression could be seen. Furthermore, the expression levels of iNOS and IL-10, which are surface markers of M1 and M2 type macrophages, respectively, were found to be significantly higher in the experimental group compared to the control tissues. qRT-PCR and ELISA results demonstrated that both IL-10 and IL-6 were expressed at significantly higher levels in the blood of the experimental group of mice. Notably, the expression of IL-10, a cytokine secreted by M2, appeared to be marginally higher. Collectively, these observations imply that TREM2 might participate in the regulatory process whereby M2 macrophages exert biological functions following *Mtb* infection, which appears to be in line with current research presumptions.

In the context of *Mtb* infection, the induction of an M1-type excessive TNF-α response has been observed to facilitate the acceleration of granuloma production and the transition of macrophages from M1 to M2-type (Redente et al., 2010; Palacios et al., 2021). Our data indicate that, during Mycobacterium smegmatis infection, macrophages may exhibit a gradual phenotypic polarization toward the M2 profile. Specifically, the expression of M1 macrophage-associated markers (TNF-α and IL-6) tended to be progressively downregulated with extended infection duration, while the transcript and protein levels of M2 macrophage-associated markers (Arg-1 and IL-10) were likely to remain elevated relative to uninfected control groups as infection persisted. As demonstrated by Western Blot analysis, the protein expression trend exhibited a high degree of concordance with the qRT-PCR results. This observation lends further support to the hypothesis that the infection-induced conversion of macrophages to M2-type macrophages is a gradual process, a finding that is in accordance with the existing literature on the subject. Emerging evidence indicates TREM2 directly binds Mtb components, subverting macrophage antimicrobial defenses to facilitate bacterial survival (Dabla et al., 2022). TREM2 expression may exhibit temporal dynamics-low during the early stage of infection but potentially increasing alongside the production of anti-inflammatory cytokines. A subsequent peak in TREM2 expression might be observed, followed by a gradual decline, though a certain level of high expression could be maintained to potentially contribute to the host’s defense against infection. Collectively, these observations may imply that TREM2 has the potential to play a significant role in the body’s resistance to TB infection.

As Turnbull et al. (2006) reported, TREM-2 binding to its ligands resulted in the inhibition of macrophage activation and the secretion of pro-inflammatory cytokines, including TNF-α and IL-6. Furthermore, TREM-2 expression may be significantly increased in the cell membranes of M2-like macrophages, while it might be almost absent in the membranes of M1-like macrophages. The observation that the expression trends of TREM2 and M2 macrophage markers are largely consistent could prompt further investigations into TREM2 and M2 macrophages. To further verify our hypothesis, human monocyte-derived THP-1 cells might be induced to undergo further differentiation, which could potentially generate M1 and M2-like macrophages. Through qRT-PCR and western blot experiments, the expression levels of TREM2 mRNA and protein might be more prominent in infected M2-like macrophages than in infected M1-like macrophages. In the later stage of infection, the upregulation of TREM2 might potentially promote the polarization transition of M2 macrophages.

Macrophage polarization was assessed using the canonical markers iNOS, Arg-1, IL-6 and IL-10, which are well-established for distinguishing M1/M2 phenotypes in mycobacterial infection research. Although additional markers (CD86, CD206, IL-12, TGF-β) could further refine polarization profiling, the selected core markers showed consistent expression trends and significant statistical differences, adequately reflecting macrophage polarization status and functional properties. Future work will adopt an expanded marker panel to enable more comprehensive characterization of macrophage phenotypic switching and immune regulatory mechanisms.

In summary, our findings may suggest that *Mycobacterium smegmatis* infection could be associated with an increase in TREM2 expression, which might in turn contribute to the phenotypic remodeling of macrophages. It could be hypothesized that TREM2 might play a role in the process whereby M2-like macrophages exert their biological functions during *Mtb* infection, and there may potentially be a reciprocal relationship between TREM2 and M2-like macrophages.

Further validation of this relationship will be performed in future studies with additional time points and more biological replicates. In addition, this study lacks sufficient functional assays; thus, our conclusions are primarily correlative, with relatively limited mechanistic interpretation. *Mycobacterium smegmatis* was adopted as the *in vitro* model in this work due to its fast growth rate, simple culture conditions, low biosafety risk and convenient experimental manipulation, making it suitable for preliminary exploration of the interaction between mycobacteria and macrophages. Nevertheless, we recognize its inherent limitations in investigating the authentic pathogenic mechanisms of *Mycobacterium tuberculosis*. Compared with virulent *Mtb*, *Mycobacterium smegmatis* cannot fully recapitulate the biological features of tuberculous granuloma formation. Accordingly, further verification using a virulent *M. tuberculosis* infection model is warranted in subsequent research to validate the translational implication of our current findings.

## Data Availability

The raw data supporting the conclusions of this article will be made available by the authors, without undue reservation.
